# Correlation of T2* relaxation times of the retropatellar cartilage with tibial tuberosity–trochlea groove distance in professional soccer players

**DOI:** 10.1038/s41598-020-72299-7

**Published:** 2020-09-18

**Authors:** Kai-Jonathan Maas, M. Warncke, C. Behzadi, G. H. Welsch, G. Schoen, M. G. Kaul, G. Adam, P. Bannas, F. O. Henes

**Affiliations:** 1 Department of Diagnostic and Interventional Radiology and Nuclear Medicine, Center for Radiology and Endoscopy, University Medical Center Hamburg-Eppendorf, Martinistrasse 52, 20246 Hamburg, Germany; 2 UKE Athleticum-Center for Athletic Medicine, University Medical Center Hamburg-Eppendorf, 20246 Hamburg, Germany; 3 Department of Medical Biometry and Epidemiology, University Medical Center Hamburg-Eppendorf, 20246 Hamburg, Germany

**Keywords:** Anatomy, Musculoskeletal system, Cartilage

## Abstract

The tibial tuberosity–trochlear groove (TT–TG) distance is a radiographic measurement that is used to quantify malalignment of the patellofemoral joint (PFJ) in cross-sectional imaging. There is an ongoing debate about the impact of the TT–TG-distance on lateral patellar instability and the initiating of cartilage degeneration. In this prospective study, the association of T2* relaxation times and TT–TG distances in professional soccer players was analyzed. 36 knees of 18 professional soccer players (age: 21 ± 2.8 years) were evaluated. Participants underwent knee MRI at 3 T. For qualitative image analysis, fat-saturated 2D PD-weighted Fast Spin Echo (FSE) and T1-weighted FSE sequences were used. For quantitative analysis, T2* measurements in 3D data acquisitions were performed. In a qualitative analysis there was no structural cartilage damage and no abnormalities of the patellar and trochlea shape. The highest T2* values (26.7 ± 5.9 ms) were observed in the central compartment of the patella. The mean TT–TG distance was 10 ± 4 mm (range 3–20 mm). There was no significant correlation between TT–TG distance and T2* relaxation times in all three compartments of the retropatellar cartilage. Our study shows that so long as patellar and trochlear morphology is normal, TT–TG distance alone does not affect the tissue structure of the retropatellar cartilage in professional soccer players.

## Introduction

The tibial tuberosity–trochlear groove (TT–TG) distance is a radiographic measurement that is used to quantify malalignment of the patellofemoral joint (PFJ) in cross-sectional imaging^1,2^. The position of the tibial tuberosity in relation to the trochlear groove is important for the inferolateral force vector of the patella^3^. Concerning patellar instability, an elevated TT–TG may cause impaired function^4^ and early cartilage degeneration^5,6^.

Cross-sectional imaging such as computed tomography (CT) or magnetic resonance imaging (MRI) is used for the measurement of TT–TG distance. It is accepted as a reference in the assessment and treatment of patellofemoral disorders^7–9^. Normal values for TT–TG distance reported in the literature show a high degree of variability (4.9–11.6 mm)^9–12^. Using an open-configuration MRI scanner, it has been shown that the TT–TG distance is affected depending on knee positioning and weight bearing^13,14^.

Among other geometric measures (e.g. trochlea dysplasia, patella alta or torsional deformities), lateral malposition of the tibial tuberosity is regarded as one important risk factor for femoropatellar instability. Therefore, a pathologic TT–TG distance is an indication for surgical therapy to correct malalignment within the PFJ^15,16^. There are different thresholds between 15 and 20 mm suggested as an indication for surgical intervention^8^. However the use of the TT–TG as criteria alone has been debated and there are also different values given for the “normal” population^17^.

Several studies proved an association of patellofemoral maltracking and a higher risk of cartilage damage^18,19^. With MRI as the preferred imaging technique for cartilage assessment Falkowski et al. found cartilage edema in the lateral retropatellar facet is associated with patellar instability^18^. In another population group with no structural degenerative changes of the cartilage, the patellar edema in the lateral facet was also considered as a precursor of osteoarthrosis in the lateral compartment of the PFJ^19^.

Recently developed T2-mapping techniques allow the subtle cartilage changes to be assessed before morphologic changes are present^20^. Previous studies have demonstrated that T2 values are reflective of subtle changes in water content and orientation of collagen fibers in knee cartilage; these indicators are signals of cartilage degradation^21^ and correlate with osteoarthritis severity^22^. T2* relaxation times reveal additional information related to local field susceptibility effects, and these may be more sensitive to changes in tissue composition compared with T2 relaxation times^23^.

Previous studies demonstrated excellent reproducibility of the T2* measurements in the cartilage of the knee and ankle joint^24,25^.

In a recent study, T2* measurements revealed that alterations in cartilage composition are associated with early osteoarthritis^26^.

In a recent study, Schenk et al. demonstrated feasibility of quantitative MRI to detect regions at risk for osteoarthritis in the femorotibial knee joint of young and healthy professional soccer players^27^. Knowing these regions can help to optimize training and prevention programs and to minimize chronic stress on vulnerable areas of the knee cartilage in young elite athletes^27^. Professional soccer players are at especially high risk of developing osteoarthrosis, often with onset even at young age, and the retropatellar cartilage is frequently affected before the tibiofemoral joint^28^. Besides traumatic knee injuries cumulative stress by kicking with a partially flexed knee and a constitutional malalignment of the PFJ are considered as potential initiators of patellofemoral cartilage degeneration^29^.

The purpose of our study was to evaluate the retropatellar cartilage using quantitative MRI in a cohort of young professional soccer players and to correlate T2* relaxation times with TT–TG distances. Using quantitative T2* mapping as a noninvasive assessment tool, we aimed to elucidate the potential impact of the TT–TG distance on compositional alterations of the retropatellar cartilage in elite athletes.

## Results

### Qualitative image analysis

According to the modified Outerbridge score, all 36 knees of the 18 participants revealed a score of 0 as there were no morphological signs of cartilage degeneration. All athlete’s scans revealed a normal patella and trochlea shape. There were eleven players with a patella shape of grade 1 and 25 grade 2 according to Wiberg. In terms of the trochlea shape, six knees were ranked with Hepp grade 1 and 30 classified Hepp grade 2. The Insall–Salvati–Index was in the range between 0.8 and 1.2 with a mean of 1.07 ± 0.15. All patients showed intact joint cartilage without morphological cartilage lesions in the PFJ. The mean trochlear depth was 7 mm (range 5–9 mm). The mean TT–TG distance was 10 ± 4 mm (range 3–20).

### Quantitative image analysis

The highest full-thickness T2* relaxation times were noted in the central compartment with 26.7 ± 5.9 ms and the lowest T2* relaxation times were registered in the lateral compartment with 25.1 ± 5.9 ms. For the medial compartment T2* times were assessed with 25.9 ± 5.9 ms. No significant difference was observed comparing the predefined compartments of the retropatellar cartilage (p = 0.16).

**Figure 1:**
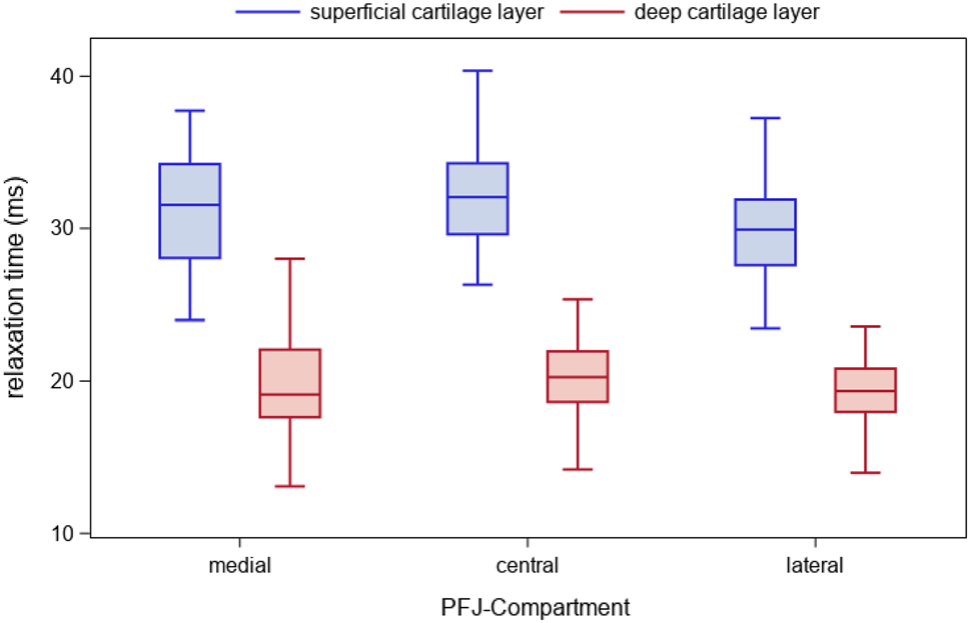
T2* relaxation times for the three predefined compartments in the deep (red) and superficial layers (blue) of the cartilage in the PFJ compartment.

T2* relaxation times were significantly higher in the superficial layers (31.3 ± 2.5 ms; range 32.5–59.4 ms) compared to the deep layers (20.5 ± 2.5 ms; range 4.6–27.8 ms) (p < 0.001). For detailed results of the three compartments and both layers see Table 1 and Fig. 1.

**Table 1:** Distribution of T2* relaxation times (range, mean) in all three compartments of the retropatellar cartilage.

Compartment	Cartillage layer	Mean T2* ± SD (ms)	T2* range (ms)	P-value
Medial	Superficial	31.2 ± 2.5	26.2–37.2	< 0.001
	Deep	20.5 ± 2.5	15.4–26.4	
Central	Superficial	32.1 ± 2.5	27–38.1	< 0.001
	Deep	21.3 ± 2.5	16.2–27.3	
Lateral	Superficial	30.6 ± 2.5	25.5–36.5	< 0.001
	Deep	19.8 ± 2.5	14.7–25.7

For the repeated quantitative measurements in five randomly selected examinations (60 ROIs), the intraclass correlation coefficient of the T2* values was 0.99 [95% confidence interval (CI) 0.9–1.0, P-value < 0.001].

**Figure 2:**
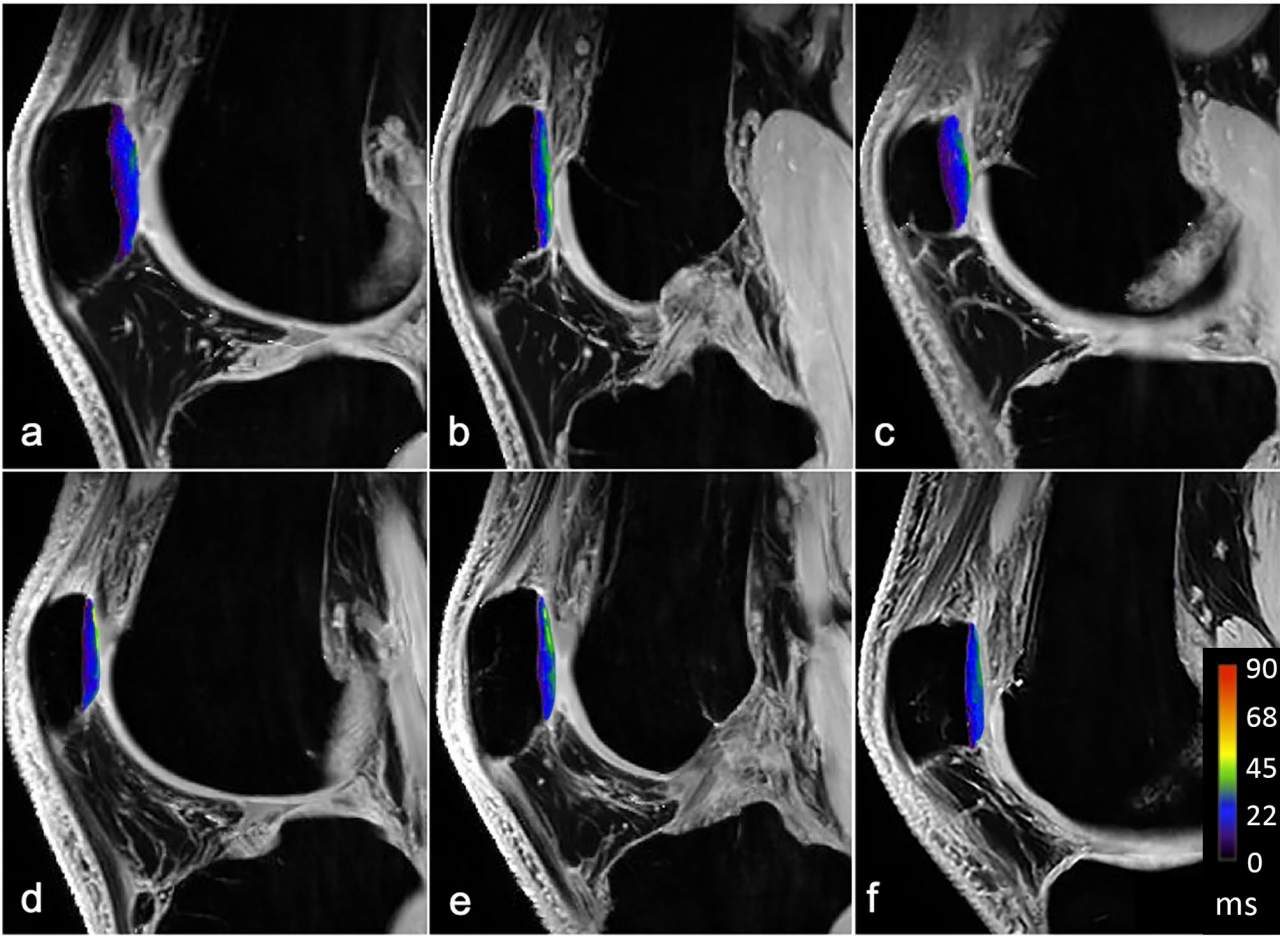
Color maps of the lateral (**a**, **d**), central (**b**, **e**), and medial (**c**, **f**) compartment of the retropatellar cartilage in a patient with low TT–TG-distance (3 mm, **a**–**c**) and a patient with a high TT–TG-distance (20 mm, **d**–**f**).

**Figure 3:**
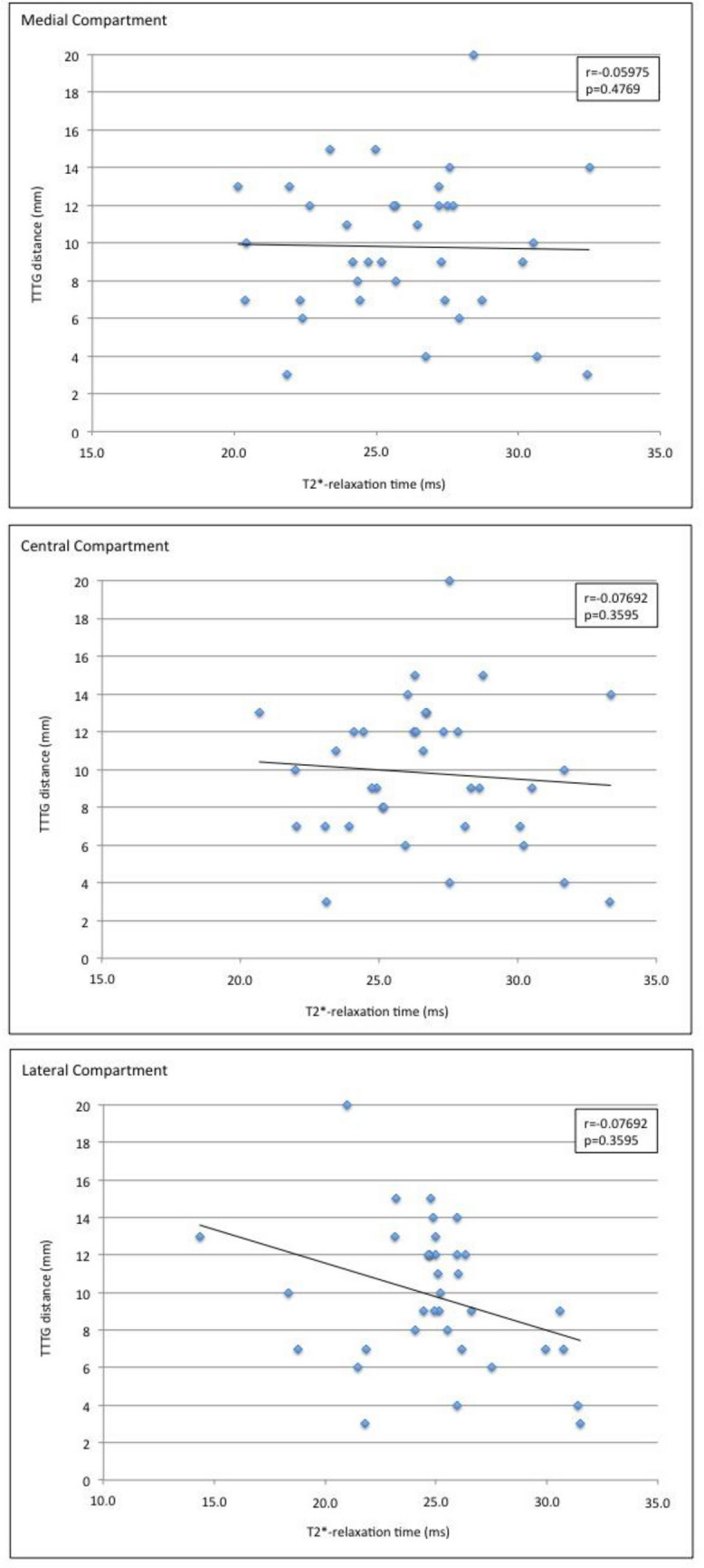
Correlation analysis between T2* relaxation times of retropatellar cartilage and TT–TG distances.

There was no significant correlation between the TT–TG distance and full-thickness T2* relaxation times in the retropatellar cartilage (r = − 0.05975; p = 0.4769 for medial compartment, r = − 0.07692; p = 0.3595 for lateral compartment and r = − 0.07692; p = 0.3595 for central compartment) (Figs. 2 and 3).

## Discussion

In our study we performed quantitative magnetic resonance (MR) analysis of the retropatellar cartilage in young professional soccer players and analyzed if there is an association of T2* relaxation times and TT–TG distances. There was no significant increase of T2* values as an indicator for early degenerative changes in correlation with higher TT–TG distances.

T2* mapping is regarded as a potential biomarker to detect early degenerative changes in cartilage. Several studies have implemented this technique to investigate baseline T2* values in healthy subjects and T2* value changes in osteoarthrosis^23,30–32^.

In our study, we found higher T2* values in the superficial layer (mean: 31.3 ± 3.8 ms; range 22.6–52.2 ms) in comparison to the deep layer (mean: 20.5 ± 4.2 ms; range 18.3–41.1 ms) of the retropatellar cartilage. These findings are in line with observations in previous studies with higher T2* values in the superficial layer in comparison to the deeper layer of the cartilage. Bittersohl et al. determined T2* values in healthy knee joint cartilage and found mean T2* values of 31.4 ± 4.1 ms in the superficial layer and of 25.3 ± 3.4 ms in the deep cartilage zones^30^. The mean full-thickness T2* value of the retropatellar cartilage in our study was 28.3 ± 3.5 ms, which is slightly lower than reported in a 3 T-study by Pai et al. (31.6 ± 4.5 ms) who assessed T2* values in the patellar cartilage of healthy adult volunteers^33^. This difference might be explained in part by the association between T2* values and age. In a recent study Tsai et al. found an age-related increase in T2* values in knee cartilage^26^.

We observed the highest full-thickness T2* relaxation times in the central compartment of the cartilage compared to the medial and lateral facets, however without reaching a statistically significant difference. Thuillier et al. showed a significant difference in T1rho values between the medial and lateral facet in patients with patellofemoral maltracking. Considering this previous work, a significant difference of T2* relaxation times in the cartilage would have been expected by the comparison of the medial and lateral facet in patients, if patellofemoral maltracking due to elevated TT–TG-distances would have been present^20^.

When comparing studies that deal with the assessment of the TT–TG distance, it is important to note which measurement technique was used and in which knee positioning the TT–TG measurement was performed. We used a measurement technique which is routinely performed in cross sectional imaging throughout the literature^9,34^. It has been argued that the position regarding the degree of knee flexion is not specified in most studies, although TT–TG distances depend on knee positioning^17^. In our study population all MR scans were performed standardized in a dedicated knee coil that allows knee positioning only in 5°–10° flexion. According to the study by Dietrich et al. who reports, that the TT–TG distance increases by approximately 5 mm, when the measurement is performed in full extension and then compared to 15° flexion, we may assume that some of the measurements might be underestimated compared to measurements in full extension^17^. We found a wide range of TT–TG-distances (3–20 mm) in our study collective. There was a proportion of athletes with TT–TG distances above 10–15 mm, which is regarded as pathological according to literature^7,35,36^. Our results are in line with a previous study by Balcarak et al. who found elevated TT–TG distances in about 10% of healthy individuals^7^. These observations suggest that elevated TT–TG distances are frequently found in the healthy population without clinical significance on the knee joint. However, at this point, referring only to cross-sectional studies, we still cannot certainly rule out that athletes with pathological TT–TG distances are at a higher risk of developing osteoarthritis in the later course of the career.

Our study has important clinical implications. A higher incidence of knee osteoarthritis is reported in athletes in comparison to the general population, especially in sports like soccer with high load bearing of the knee^37,38^. As we had a very specific patient population with young professional soccer players without any morphological cartilage defects we were able to assess the possible impact of an increased TT–TG distance. Within PFJ alignment, the position of the tibial tubercle plays a vital role, contributing to the inferolateral force vector on the patella^9,39^. During knee movement the PFJ contact area is shifted laterally creating further mechanical stress on the lateral patellar and trochlear facets^40,41^. Consequently, due to developmental or acquired alterations in the anatomy and congruence of the PFJ, patients may develop cartilage lesions and subsequent osteoarthritis^42^.

We found no indications for a potential risk of early cartilage degeneration in soccer players with increased TT–TG-distances. This observation is in line with previous studies. Tsavalas et al. found no significant differences of TT–TG distances between patients with osteoarthritis and controls^36^. In a comparative study between the symptomatic and asymptomatic knees of patients with recurrent unilateral patellar instability there was also a lack of significant differences in TT–TG distances^43^. In contrary, in a more recent study, TT–TG distance was considered as critical factor for the severity of patellofemoral osteoarthritis and authors suggested that external rotation of the lower limbs might induce patellofemoral osteoarthritis^44^. However, the correlation of increased TT–TG distance in this study was found especially for end-stage disease and not for initial cartilage degeneration.

To the best of our knowledge, this is the first study that aims to provide a more distinct view on the TT–TG distance and its effect on cartilage composition in young professional athletes. As we performed standardized MR examinations in a young healthy cohort of professional athletes we minimized differences in loading conditions and diurnal variations in articular cartilage and thereby limiting a certain degree of inconsistency in the T2* measurements.

Contrary to previous studies we enrolled a unique population of young professional soccer players who undergo high intensity of daily training with heavy weight bearing of the knee joint. This study population is at extreme high risk of the development of osteoarthritis, especially of the PFJ and with initiation of osteoarthritis at a young age. Furthermore, we performed quantitative MRI of the cartilage in our study. T2* mapping can be seen as a marker for tissue alterations at a very early stage as T2* changes occur before the onset of clinical subjective or objective symptoms^26^.

Our study has some limitations. First, we focused on a collective of healthy young athletes without symptoms. However, by doing so, we minimized potential adverse influences such as age and the risk of undiagnosed cartilage lesions. In return, the T2* values obtained in this study may not be representative for an older control group, despite the fact that young people would achieve the greatest benefit from early cartilage damage diagnosis and corresponding intervention. For quantitative analysis in T2* mapping, we performed manual ROI analyses in this study, which may certainly create an operator-dependent bias. As a solution for future longitudinal/follow-up studies to compare cartilage T2* values at different time points, semiautomated image registration systems may be of great benefit. Another limitation of our study was a lack of soccer players with anatomical risk factors of patellar instability such as trochlear dysplasia or patella alta. Future studies including athletes with those risk factors are needed to elucidate the impact of the patellar and trochlear morphology on cartilage degeneration in professional soccer.

Finally, due to the strict inclusion criteria with the involvement of only young professional soccer players, our study was based on a relatively small number of patients.

In conclusion, in our population of young professional healthy athletes there was no correlation of T2* relaxation times in the retropatellar cartilage and TT–TG distance.

Our study shows that so long as patellar and trochlear morphology is normal, TT–TG distance alone does not affect the tissue structure of the retropatellar cartilage in professional soccer players.

Further longitudinal studies including athletes of different age ranges and with different trochlear morphologies are needed to further evaluate the value of TT–TG as a biomarker for the prediction of cartilage degeneration.

## Material and methods

The study was approved by the local Ethical and Research Committee in Hamburg, Germany “Ärztekammer Hamburg” (PV4761) and conducted under compliance of the local Institutional Review Board in accordance with the Helsinki Declaration of 1975 as revised in 1983. All participants gave written informed consent and the data approved by the Ethical committee were anonymized and de-identified for analysis.

### Study population and inclusion/exclusion criteria

Between January and July 2018, we enrolled 18 professional male soccer players of an elite sports academy of the highest national soccer league (mean age: 21 ± 2.8 years, range 19–29 years). MRI of all players for both knees was performed during preseason as part of a medical suitability examination. Thus 36 MR examinations were performed in total. All players were examined in the morning and after a resting period of at least 24 h. For each participant, the study date was chosen individually to exclude any effects of high cartilage loading related to training or a preseason match.

Exclusion criteria were acute injury or history of major injury of the knee (e.g., fracture, prior surgery), systemic inflammatory disease (e.g., rheumatoid arthritis), or major sport event during the week prior to MRI. All participants had a free range of motion in the joint and did not report any knee pain at the time of investigation.

### MRI acquisition

A resting period of 30 min in the supine position prior to image acquisition was kept constant for all participants in order to reduce any possible influence of daily physical loading on quantitative measurements.

MRI was performed using a 3 T MR system (Ingenia, Philips, Best, The Netherlands). A dedicated 8-channel knee coil (dStream, Philips, Best, The Netherlands) was used with maximum knee flexion of 5°–10°. For qualitative image analysis, a fat-saturated 2D PD-weighted Fast Spin Echo (FSE) and T1-weighted FSE sequence was used. For quantitative image analysis, a sagittal 3D T2* weighted Gradient Echo sequence (GRE) was applied including the following imaging parameters: 22 echoes with TE: 4.6–53.6; TR: 150 ms; matrix 320 × 320; FOV 160 × 160 mm; slice thickness of 1 mm resulting in a resolution of 0.5 × 0.5 × 1 mm^3^; scan time 6:58 min. *T*_2_* mapping was performed by fitting a monoexponential function A*exp(− TE/*T*_2_*) to the multi-echo data using an in-house quantification plugin (qMapIt) extending ImageJ (National Institutes of Health, Bethesda, MD)^45^. Overall scan time was 17:13 min.

### Qualitative image analysis

All MR data sets were analyzed in consensus by two radiologists with three and twelve years of experience, respectively in musculoskeletal radiology using a commercially available post processing workstation (Extended Brilliance Workspace, Version 2.0, Philips Healthcare, Best, The Netherlands).

For all participants included in this study, the modified Outerbridge score was assessed for semi-quantitative grading of retropatellar chondral lesions^46^. In all examinations, the patella and the trochlea shape were assessed using the classification of Wiberg and Hepp, respectively. The Wiberg patellar shape is determined by the size of the medial and lateral facets^18^. The trochlear shape is determined by the symmetry of elevation of the medial and lateral trochlear surfaces^47^. In both classifications type 3–5 are considered abnormal, predisposing patients to early osteoarthrosis^18,47^. Moreover, the Insall–Salvati Index was determined to assess patella alta or bacha.

The trochlear depth was assessed in the transverse MR image, 3 cm above the femorotibial jointspace according to a measurement technique described elsewhere^48^.

If divergent results were noted, both radiologists consensually revised images until final consensus was reached.

### TT–TG distance measurements

**Figure 4:**
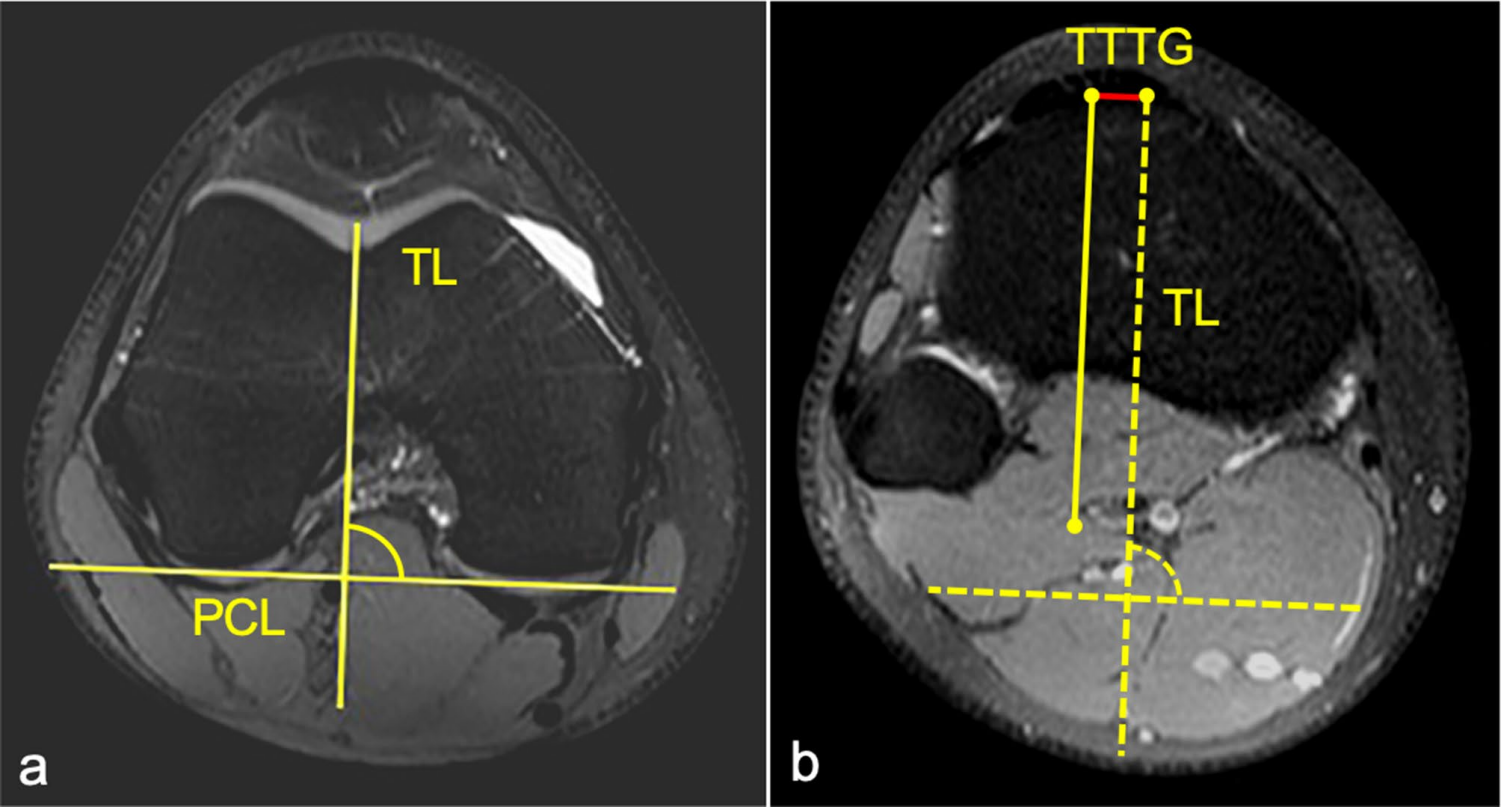
Determination of the TT–TG distance using fat suppressed PD-weighted images in transversal orientation. First, the most proximal MR image slice with full cartilage coverage of the trochlear groove is chosen to assess the trochlear groove, and the posterior condylar line is drawn tangential to the chondral border of the posterior condyles of the femur. Further, the TL is drawn perpendicular to posterior condylar line (**a**). Secondly, TL will be transferred to most cephalad image in which the patellar tendon is fully in contact with the tibial tubercle. The center of the tibial tubercle is marked at the midpoint of the patellar tendon on this image. The final TT–TG distance will be taken as the perpendicular distance from the center of the tibial tubercle to the transferred TL (**b**).

For measuring the TT–TG distance, patient’s knees were in 5°–10° flexion, their quadriceps was relaxed, and their feet were placed in a neutral rotation prior to the MRI scan. The TT–TG measurement was adopted to the technique described by Schoettle et al.^34^. The most proximal transverse image that depicted a complete cartilaginous trochlea was used to determine the deepest point within the trochlear groove. A line was drawn through the deepest point of the trochlear groove, perpendicular to the posterior condyle tangent. On the most proximal image of the insertion of the patellar ligament and the tibial tuberosity, a second line was drawn in parallel to the trochlear line (TR) through the most anterior portion of the tibial tubercle. The distance between the two lines determines the TT–TG distance (Fig. 4).

### Quantitative image analysis (T2* measurements)

**Figure 5:**
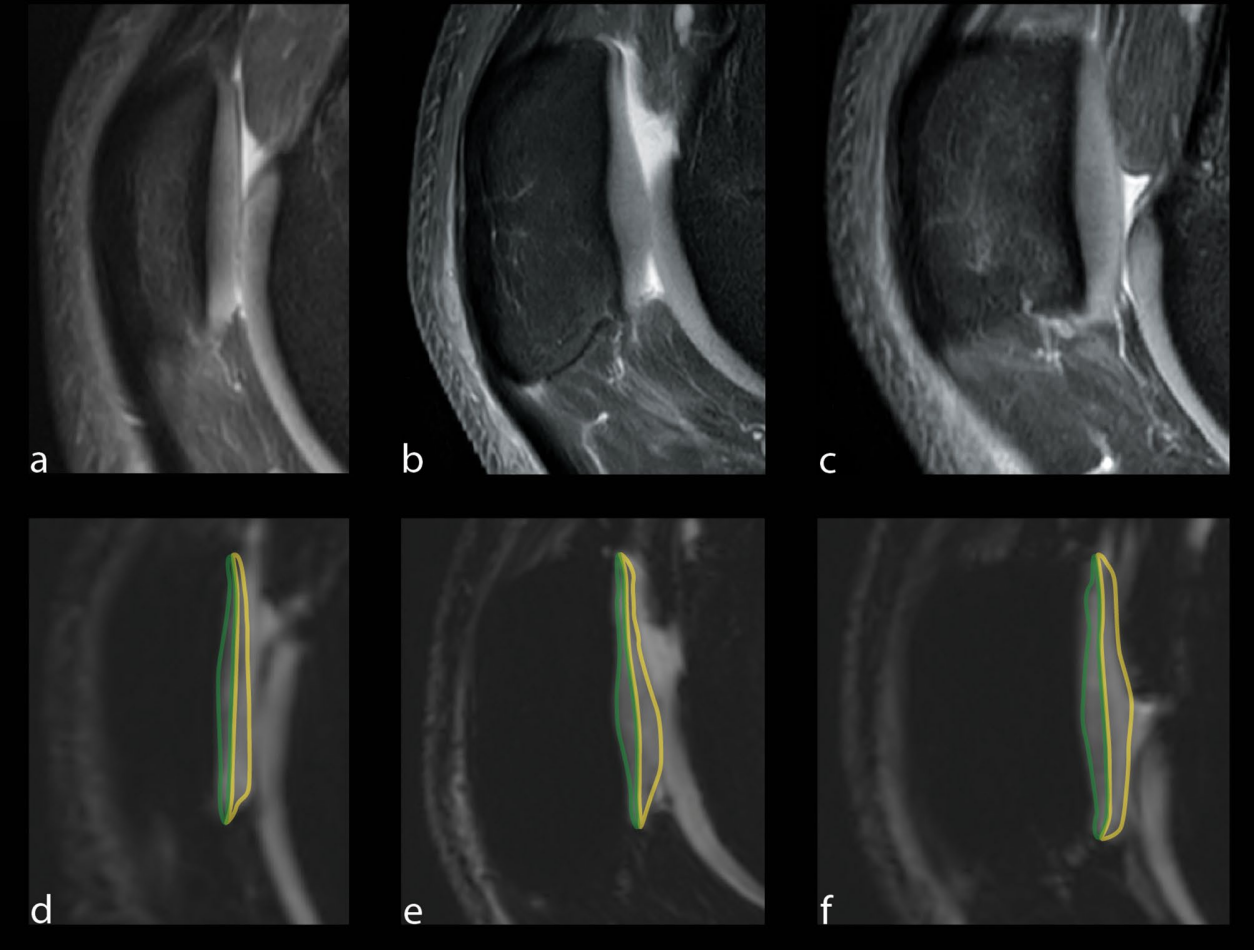
The predefined areas of the retropatellar cartilage for T2* measurements with the corresponding PD weighted images for morphological orientation (**a**–**c**). T2* measurements were performed on sagittal images in the deep (region of interest in green) and superficial (region of interest in yellow) layer of the retropatellar cartilage. Measurement were performed in the lateral (**d**), central (**e**) and the medial (**f**) compartment of the joint space.

The patella was evenly divided into a lateral, central, and medial compartment for quantitative analysis^49^. The trochlea sulcus served as the anatomic landmark for the central compartment. The total numbers of slices covering the retropatellar cartilage were assessed both to the lateral and medial borders of the patella. Quantitative image analysis was performed on the two most central consecutive slices of each compartment. The articular cartilage of the patella was outlined without any subchondral bone and synovial fluid. A specific bone–cartilage threshold was set for each participant to automatically remove possible included cortical bone pixels within the ROIs. Each compartment of the cartilage was evenly divided into two layers, representing superficial and deep layers (Fig. 5).

In each compartment, ROI analysis was performed on the two most central consecutive slice images of each compartment, using a dedicated software application (ImageJ). The average of the T2* values of the consecutive images was defined as the T2* value of the ROI, as performed elsewhere^49^. The voxel count of the ROIs was documented (138.3 ± 18.81 voxels [minimum 113, maximum 186]) as well as the number of voxel layers in each cartilage layer (mean 6; range 5–7).

In the 36 knees evaluated, a total of 432 ROIs were manually placed. All measurements were performed in consensus by both radiologists.

To assess the reproducibility of our results, the T2* measurements were repeated by the same two readers in five randomly selected knees of our study population with a time delay of 6 months.

### Statistical modeling

Since there are 24 measurements per patient, an independence of measurements cannot be assured. To account for this cluster structure at the patient level we estimated a mixed random effects model to examine the effect of the TT–TG angle on the T2* relaxation time. The model was calculated with the T2* relaxation time as a dependent variable, TT–TG angle, superficial vs deep surface as well as the three investigated areas (medial, central, lateral) as fixed effects and patient ID as random effect.

To examine the importance of the division into the three areas for the effect of the TT–TG angle on the relaxation time, a likelihood ratio test was performed to compare the goodness of fit of both models with and without the three areas, respectively.

To analyze the intra-reader reliability of the measurement technique (five MR studies were analyzed twice by the same readers) we used the intraclass correlation coefficient in a two-way mixed model with single measures.

All statistical analyses were performed with the statistical software SAS, version 9.4. The standard level of significance used to identify statistically significant effects was set to 0.05.
